# Virus Infection of a Freshwater Cyanobacterium Contributes Significantly to the Release of Toxins Through Cell Lysis

**DOI:** 10.3390/microorganisms13030486

**Published:** 2025-02-22

**Authors:** Victoria Lee, Isaac Meza-Padilla, Jozef I. Nissimov

**Affiliations:** Department of Biology, University of Waterloo, 200 University Ave. West, Waterloo, ON N2L 3G1, Canada; v49lee@uwaterloo.ca (V.L.); imezapadilla@uwaterloo.ca (I.M.-P.)

**Keywords:** harmful algal bloom, *Microcystis aeruginosa*, microcystin, cyanophage, virus infection

## Abstract

Toxic algal-bloom-forming cyanobacteria are a persistent problem globally for many aquatic environments. Their occurrence is attributed to eutrophication and rising temperatures due to climate change. The result of these blooms is often the loss of biodiversity, economic impacts on tourism and fisheries, and risks to human and animal health. Of emerging interest is the poorly understood interplay between viruses and toxic species that form blooms. This is because recent studies have suggested that viruses may exacerbate the harmful effects of these blooms by contributing to the release of toxins into a dissolved phase upon cell lysis. However, to date, there is no experimental evidence that explicitly implicates viruses in microcystin release. Here, we show experimentally that a virus infection of the toxin-producing, harmful, algal-bloom-forming cyanobacterium *Microcystis aeruginosa* results in a 4-fold increase in the toxin microcystin-LR two days post-infection (dpi). We also show that the concentrations of microcystin remain high after culture discoloration and host cell lysis. Collectively, our results directly implicate viruses as major contributors to microcystin release from cyanobacteria and emphasize the importance of taking viruses into account in predictive models and in the assessment of water quality and safety.

## 1. Introduction

Aquatic habitats are essential ecological components of the biosphere. They provide drinking water and raw materials for agriculture and livestock, are foundations for fisheries and places for recreation [1,2], and support cultural eco-system services [3]. However, high concentrations of nutrients and elevated temperatures [4,5,6,7,8] puts them at an increased risk of harmful algal blooms (HABs), many of which are produced by cyanobacteria. HABs induce hypoxia due to an excessive increase in respiration during decomposition, leading to an increase in mortality and the loss of biodiversity in aquatic organisms [9,10]. Many HAB-forming cyanobacteria produce potent toxins, such as microcystins, nodularins, and cyanopeptolins, that cause liver damage and affect the nervous system [11,12,13], as well as taste and odour compounds, such as geosmin and 2-methilisoborneol (2-MIB), that cause recurring problems in certain drinking water reservoirs [14]. Additionally, emerging research suggests that cyanobacteria may be involved in global methane production processes [15], making them of further relevance to aquatic biogeochemistry and climate change.

Some of the most notorious HAB-producers are species of *Microcystis* and *Nodularia*, globally distributed freshwater and brackish water cyanobacteria, respectively. In their natural environment, cyanobacteria are associated with other microbes, including viruses [16,17,18,19]. Recent studies hint at the fact that the harmful effects of toxins being released from HAB-producers may be exacerbated by cyanophage infections (i.e., viruses that infect cyanobacteria). For example, virus infection experiments of *Nodularia spumigena* empirically demonstrated a significant increase in the hepatotoxin nodularin in the culture medium during the infection [20]. Similarly, viral activity in Western Lake Erie during *Microcystis*-dominated HABs was implicated through sequencing approaches as an important contributor to the increase in microcystins, and in shifting those toxins from a particulate to a dissolved fraction [21,22]. However, contrary to nodularins, there is no empirical evidence to date that directly implicates cyanophages experimentally in the release of microcystins. Indeed, while viruses are the most abundant biological entities in aquatic habitats [23,24,25,26,27], the degree to which they affect the production and release of toxins (and other bioactive compounds) by HAB-forming cyanobacteria, and by extension, the implications this may have on water quality and safety, are still poorly studied.

**Figure 1:**
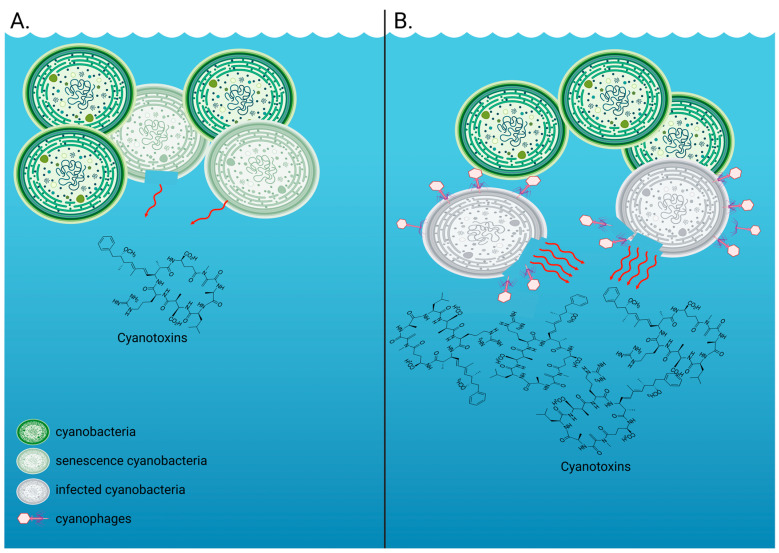
Extracellular cyanotoxin release by cyanobacteria such as Microcystis aeruginosa. (**A**) The measurable extracellular fraction of cyanotoxins in the absence of a virus infection includes toxins that are typically released upon senescence and/or cell death, with some cyanobacterial species being able to release toxins without cell rupture or death. This is what we measured in our control, uninfected M. aeruginosa NIES-298 treatments. (**B**) The measurable extracellular fraction of cyanotoxins in the presence of viruses includes intracellular toxins that are typically contained within the cyanobacterial cells and are released upon cell lysis. This is what we measured in our virus-infected M. aeruginosa NIES-298 treatments. Created with BioRender.com.

Using *Microcystis aeruginosa* strain NIES-298 and its double-stranded DNA cyanophage Ma-LMM01 [28] as an experimental model system, we performed infection experiments to characterize the temporal dynamics of extracellular microcystin-LR (Figure 1), the most toxic variant of microcystin [29], during a virus infection. We were able to empirically show elevated levels of this toxin in infected treatments after cell lysis. Those levels were much higher than 1.0 ppb, the World Health Organization (WHO)-recommended upper limit of microcystin-LR in drinking water [30]. Our present study shows for the first time that the cyanobacterial toxin microcystin-LR is indeed released in extremely high levels into the surrounding water due to virus infection of *M. aeruginosa*, levels that have clear ecological and human health implications.

## 2. Materials and Methods

### 2.1. Cyanobacterial Growth Conditions

Axenic culture stocks of *Microcystis aeruginosa* NIES-298 were purchased from the National Institute for Environmental Studies in Japan and were periodically monitored microscopically for bacterial contamination prior to our experiments. NIES-298 was cultivated in BG-11 liquid media (pH~7.5), which was purchased from the Canadian Phycological Culture Centre (https://uwaterloo.ca/canadian-phycological-culture-centre/cultures/culture-media/bg-11; access on 10 October 2024) and maintained in a light/dark cycle of 12:12 h (~50 μmol m^−2^ s^−1^) and at a temperature of 25 °C (±1 °C). The same outlined growth conditions were also used in the routine propagation of fresh viral lysates and during the ELISA experiments (see below).

### 2.2. Virus Propagation

Fresh viral lysate of the cyanophage Ma-LMM01 used in this study was obtained by infecting at an MOI of ~0.5 exponentially growing *M. aeruginosa* NIES-298 (i.e., at a cell density of ~2 × 10^7^ mL^−1^), filtering the lysed cells through a sterile Millex-HV PVDF (Millipore, Burlington, MA, USA) 0.45 µm filter (has a pore size that allows the passage of these virions, which have a tail of ~200 nm in length [31]), and storing the filtrates in the dark at 4 °C until the onset of the ELISA experiments.

### 2.3. Infectious Titre Quantification of Virus Lysate

To ensure that the freshly generated Ma-LMM01 cyanophage lysate used in our experiments contained infectious particles, we conducted most probable number (MPN) assays [32] on the lysate. Briefly, *M. aeruginosa* NIES-298 was cultivated to the late exponential phase, after which 240 μL was distributed into individual 96-well plates. Then, 10-fold serial dilutions of the viral lysate were prepared. Subsequently, 10 μL of 0.45 μm filtered media as a negative control was loaded in each well in column 1 of the 96-well plate (A1–H1), 10 μL of undiluted lysate was loaded as positive controls in each well in column 2 (A2–H2), and 10 μL of dilutions 10^−1^–10^−10^ was loaded in columns 3–12. The plates and the wells within them were visually inspected for infection (i.e., culture discoloration/loss in pigmentation relative to controls) seven days post-virus addition (i.e., culture discoloration in relation to controls). The scores (i.e., number of cleared wells per dilution) were then inputted into the Environmental Protection Agency’s most probable number (MPN) calculator (https://mostprobablenumbercalculator.epa.gov/mpnForm; access on 10 October 2024) to obtain an MPN number (i.e., the number of infectious virus particles in each lysate).

### 2.4. Cell Density Measurements

**Figure 2:**
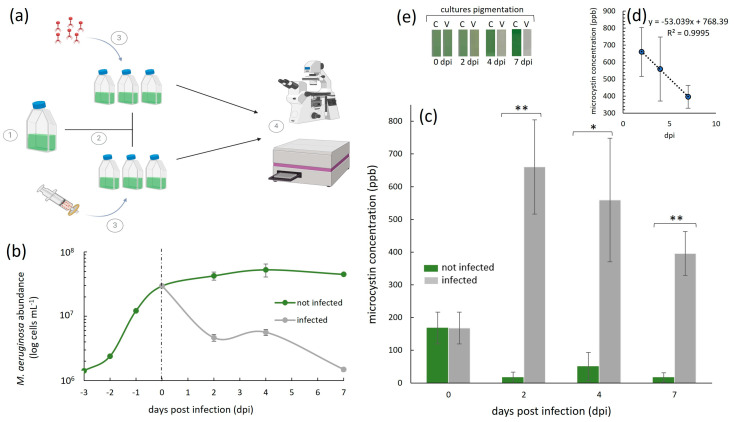
Cyanophage-infected and uninfected *M. aeruginosa* NIES-298 cyanobacterial cultures and the subsequent analysis of extracellular microcystin-LR dynamics during infection. (**a**) 1—Cyanobacterial cells were incubated until the late exponential growth phase (i.e., 2.95 × 10^7^ cells mL^−1^); 2—the culture was then split into six replicates at day 0 (dashed line in (**b**)), three of which were infected with a cyanophage Ma-LMM01 stock that was at a virus particle density of 1.35 × 10^7^ mL^−1^ and three of which were inoculated with an equal volume of a 0.02 µm filtrate of the Ma-LMM01 stock; and 4—ELISA essays and total NIES-298 cell abundance measurements were performed using spectrophotometry and haemocytometry, respectively. (**b**) *M. aeruginosa* NIES-298 growth dynamics (*n* = 3, ±SD) of Ma-LMM01-infected (grey line) and uninfected (green line) treatments up to seven days post-infection on day 0 (indicated as a dashed line). (**c**) Average (± SD, *n* = 3) extracellular microcystin-LR concentrations in parts per billion (ppb) in cyanophage Ma-LMM01-infected (dark grey bars) and uninfected (green bars) treatments. ** and * denote significant differences (*p* < 0.01 and *p* < 0.05, respectively; ANOVA) between infected and uninfected treatments at individual time points. (**d**) Average daily rate of extracellular microcystin-LR decrease in infected treatments, calculated between the highest measured concentration on day 2 and the last day of the experiment on day seven. (**e**) Culture pigmentation (photographed) of a representative triplicate treatment, which was either infected (V) or uninfected (C) by viruses, 0–7 dpi. Panel (**a**) was created with BioRender.com.

Throughout our experiments, we quantified the host cell density with a haemocytometer (0.1 mm deep) under an optical microscope (Olympus BH-2; Olympus Optical Co., Ltd., Tokyo, Japan) at the 40× magnification. For each sample, we loaded 10 µL and counted the number of *M. aeruginosa* NIES-298 cells in five squares, from which we derived an average. These counts were obtained after first heating up the cells at 60 °C for 20 s. This was performed because *M. aeruginosa* cells have gas vacuoles, which makes them float in the counting chamber/haemocytometer grid and therefore makes them difficult to count. The heating step bursts the vacuoles, allowing the cells to stay on the same plane of view while counting. The error bars in the cell abundance graphs (Figure 2b) represent ±1 standard deviation (SD) of the mean of a given treatment, set up in triplicate (*n* = 3).

### 2.5. Infection Experiments and Toxin Concentration Measurements

A 300 mL *M. aeruginosa* NIES-298 culture was incubated until it reached a cell density of 2.65 × 10^7^ cells mL^−1^ (i.e., late exponential growth phase). This culture was then split into six 40 mL cultures (Figure 2a). Three of the six cultures were infected with 2.5 mL of the freshly made Ma-LMM01 cyanophage lysate (see above), which contained 1.35 × 10^7^ mL^−1^ of infectious viruses (i.e., an MOI of ~0.5 to reduce the disruption of growth dynamics due to additions of high volumes of virus lysate). The other three served as no-virus control cultures, to which we added 2.5 mL of 0.02 µm (Whatman Anotop^TM^ 25 mm, inorganic membrane, sterile; Dassel, Germany) Ma-LMM01 cyanophage filtrate. Extracellular microcystin-LR (Adda-specific) concentration (Figure 2c) measurements were then obtained with a commercially available enzyme-linked immunosorbent assay (ELISA) kit (Enzo Life Sciences, Long Island, NY, USA) up to seven days post-virus infection (e.g., time of infection: 48 h post-infection [hpi], 96 hpi, and 168 hpi) in accordance with the manufacturer’s manual, using a BioTek Synergy LX Microtiter Plate reader (Winooski, VT, USA). Note that these measurements did not include an evaluation of the concentration of the total (cells + medium) or intracellular (within cells) microcystin-LR (e.g., we did not sonicate cells or investigate the concentration within the cells collected on filters). The error bars in the microcystin-LR plot (Figure 2c) represent ±1 standard deviation (SD) of the mean of a given treatment, set up in biological triplicate (*n* = 3). A one-way analysis of variance (ANOVA) with a significance threshold defined by a *p*-value of <0.05 was performed for each sampled time point, comparing the extracellular microcystin-LR concentration we detected in the infected and non-infected treatments. In parallel to our ELISA measurements, we obtained *M. aeruginosa* NIES-298 cell density counts (Figure 2b) as previously described.

### 2.6. Calculated Cellular Microcystin-LR Release per Cell and Estimated Degradation Rate

To calculate the concentration of microcystin-LR release per lysed cell in our experiments, we first calculated the total average increase in extracellular microcystin-LR in the first two days post-virus infection (Figure 2c). We then divided this number by the total average cell decrease (cell loss) in that same time period (Figure 2b). To estimate the daily rate of extracellular microcystin-LR decrease in our experiments throughout the 7 days, we first averaged the measured fold decrease in extracellular microcystin-LR from day 2 to day 4, and from day 4 to day 7. We then averaged the two numbers, which resulted in an average daily fold decrease of extracellular microcystin-LR for our experiments (Figure 2d). Note that these calculations do not take into account the volume in which the microcystin would become dissolved (e.g., its half-life) or other factors responsible for degradation; hence, they are estimates.

## 3. Results and Discussion

Our study involved the analysis of extracellular microcystin-LR concentrations during a virus infection (Figure 2). The starting average (*n* = 6) microcystin-LR concentration (ppb) in our *M. aeruginosa* NIES-298 cultures at the time of the virus infection on day 0 (Figure 2c) was 167.76 ppb (±48.47). In uninfected cultures, this concentration decreased from this value over days 2-7. This decrease was likely because day 0 represented the late exponential growth phase of *M. aeruginosa* NIES-298 in our setup, after which the cellular growth plateaued and subsequently halted (Figure 2b). This was expected, given that our experiments were conducted in batch cultures and did not involve additions of nutrients or CO_2_ throughout the experiments. A reduction in extracellular microcystin levels, and thus, their detection in cyanobacterial cultures that are no longer growing, has been previously shown to occur due to photolysis [33,34], light exposure that can lead to the degradation of the toxin [35], and intracellular retention, where halted cellular growth results in microcystin remaining predominantly in an intracellular state [7,36].

In the cyanophage Ma-LMM01-infected cultures (*n* = 3), the concentration of extracellular microcystin-LR remained extremely high throughout the experiment compared to the uninfected controls (Figure 2c). The highest toxin concentration (660.34 ppb, ±144) was measured two days post-infection (dpi), where it was an average of ~40-fold higher than in the respective uninfected treatments (16.41 ppb, ±17). This represents a concentration increase of nearly 4-fold in two days (i.e., total average increase of 492.58 ppm, ±96.55) from day 0. By the end of the experiment on day 7, the concentration of the extracellular toxin was still high, but decreased to 395.80 ppb (±67.28). This is interesting because additional cell lysis occurred during this time period (Figure 2b), yet the concentrations of the extracellular toxin we detected decreased from its peak on day 2 post-infection. This suggests that, although there was an initial increase in extracellular microcystin-LR in the first 2 days post-virus infection, those toxins may be readily degradable, even when further lysis continues to occur. This implies that the degradation rate in our setup may have been higher than the release due to the virus infection, especially in the later stages of the virus infection. In the absence of microbial degraders in our cultures, this degradation was likely due to photolysis, although further experimentation is required to confirm this hypothesis. Nevertheless, despite this possible degradation, the levels of this toxin were still much higher than the provisional recommended upper limit for drinking water by the World Health Organization (WHO), which, for the most common and toxic microcystin, microcystin-LR, is 1.0 ppb [30]. They were also higher than the WHO’s recommended microcystin-LR concentrations for recreational waters, which are designated as being of a moderate risk at levels of 10–20 ppb and of a high risk at levels of >20 ppb.

This initial increase was due to the lysis of 2.49 × 10^7^ cells mL^−1^ (±5.5 × 10^5^ cells mL^−1^), measured here as a decrease in the cell abundance from day 0 to 2 dpi (Figure 2b). Assuming an equal microcystin-LR release for each lysing cell due to cyanophage infection, this means that virus-induced cell lysis was responsible for the release of 1.98 × 10^−5^ ppb cell^−1^ in the first two days. And although the average measured extracellular microcystin-LR concentration remained high until the end of our experiments (Figure 2c), it appeared to decrease daily by an average of 0.53-fold (Figure 2d), to an average of 395.8 (±67.28) on day 7. This decrease is not surprising, given that it was shown previously that extracellular microcystin degrades much more rapidly than intracellular microcystin [37,38].

Extrapolating our results to the situation in situ is challenging for several reasons. First, in this work, we did not measure the toxin’s concentrations beyond day 7; hence, we cannot conclude whether the initial rate of degradation that we observed and calculated would be the same until it reaches below the levels of detection. Second, natural *M. aeruginosa* cell abundances in a bloom are normally lower than in our laboratory experiments, so the amount of toxins being released due to infection would differ (and likely would be lower). Third, cyanoHABs occur in complex environments in the presence of diverse microbial communities. Indeed, the degradation of extracellular cyanotoxins in situ was previously attributed to toxin consumers that use them as a carbon source for growth [39,40,41]—consumers that were not present in our setup due to its axenic nature. And finally, our observed host–virus–toxin dynamics do not take into account other physicochemical and biological factors that are at play in situ (e.g., temperature and pH variations, nutrient loads and the trophic status of a system, the presence or absence of grazers, host resistance to infection, the diversity and abundance of other microbes, virus decay rates, and variations in the photodegradation rate of toxins driven by diel cycles, the turbidity of the water, and the latitude of a given environment).

Nevertheless, to the best of our knowledge, this is the first time in which a cyanophage infection of a cyanotoxin producer has been used to empirically demonstrate the ability of viruses to contribute substantially to microcystin-LR release, and this is the first study to report on the amount of this toxin being released from a *Microcystis* cell in response to infection. These are important steps in beginning to think about the role of viruses in HAB formation and decline by policy makers. They are also important steps that can serve as a “springboard” for the inclusion of viruses in HAB dynamics modelling; predictive models rely on the responses of toxic species to both abiotic and biotic drivers [42].

One of the most interesting observations in this work is that we detected high levels of extracellular microcystin-LR in parallel to culture discoloration (Figure 2e) and a cyanobacterial abundance decrease due to the virus infection (Figure 2b). This reinforces the importance of actively measuring toxin levels whenever possible, rather than relying solely on cyanobacterial biomass and chlorophyll measurements, or the quantification of genes involved in toxin production. Indeed, water quality guidelines reiterate that water monitoring should be a multifaceted approach, including the use of enzyme-linked immunosorbent assays (ELISAs), liquid chromatographic methods, and protein phosphatase inhibition assays [43]. However, due to the expense of these assays and the need for specialized infrastructure and trained personnel, these more complex approaches may not always be an affordable option, especially for those in low-income or developing countries. A solution may be the use of mobile apps (e.g., the Cyanobacteria Assessment Network Application app developed by the United Stares Environmental Protection Agency and the Bloomin’ Algae app developed by the UK Centre for Ecology and Hydrology, to name a few) that depend on satellite data to detect and map the location of cyanobacterial blooms, helping decision makers to rapidly detect potential HABs. As we have shown, water toxicity and the cyanobacterial biomass do not always correlate (Figure 2b,c,e). Therefore, tools that rely on the detection of water discoloration as an indicator of water safety are not reliable, especially in settings that are characterized by high viral activity (resulting in a low cyanobacterial biomass).

## 4. Conclusions

To the best of our knowledge, this is the first study in which a cyanophage infection is specifically implicated in the release of high levels of microcystin-LR—levels that remain high even after cyanobacterial host lysis, a loss in water coloration, and the likely onset of degradation processes. Our study focused on the effects of a cyanophage that is specific to a cyanobacterial species, and is hence most relevant to situations where the bloom-forming species is *M. aeruginosa.* The in situ impact of viruses with a wider host range is likely to be different on the hosts that they infect, their dominance in a system, and the dynamics of toxins. Nevertheless, our findings have real-world implications for aquatic habitats because they re-emphasize the risk of ignoring the role of virus infections of HAB formers. The current HAB-monitoring guidelines lack a specific reference to the immediate and long-term impacts of viruses, even though they are clearly important actors. This work also has specific implications for those that study the taste and odour compounds in cyanobacteria and the potential contributions of cyanobacteria to climate change through the release of methane. This is because viruses likely play an equally important role in the release of nuisance compounds and greenhouse gases, respectively.

## Data Availability

Data supporting the conclusions of this article can be made available by the authors upon request.
